# Dual Temporal Scale Convolutional Neural Network for Micro-Expression Recognition

**DOI:** 10.3389/fpsyg.2017.01745

**Published:** 2017-10-13

**Authors:** Min Peng, Chongyang Wang, Tong Chen, Guangyuan Liu, Xiaolan Fu

**Affiliations:** ^1^Chongqing Key Laboratory of Non-linear Circuit and Intelligent Information Processing, Southwest University, Chongqing, China; ^2^School of Electronic and Information Engineering, Southwest University, Chongqing, China; ^3^Institute of Psychology, University of Chinese Academy of Sciences, Beijing, China

**Keywords:** micro-expression recognition, deep learning, optical flow, convolutional neural network, feature fusion

## Abstract

Facial micro-expression is a brief involuntary facial movement and can reveal the genuine emotion that people try to conceal. Traditional methods of spontaneous micro-expression recognition rely excessively on sophisticated hand-crafted feature design and the recognition rate is not high enough for its practical application. In this paper, we proposed a Dual Temporal Scale Convolutional Neural Network (DTSCNN) for spontaneous micro-expressions recognition. The DTSCNN is a two-stream network. Different of stream of DTSCNN is used to adapt to different frame rate of micro-expression video clips. Each stream of DSTCNN consists of independent shallow network for avoiding the overfitting problem. Meanwhile, we fed the networks with optical-flow sequences to ensure that the shallow networks can further acquire higher-level features. Experimental results on spontaneous micro-expression databases (CASME I/II) showed that our method can achieve a recognition rate almost 10% higher than what some state-of-the-art method can achieve.

## Introduction

Facial expression plays an important role in people's daily communication and emotion expression. Typically, a full facial expression last from 1/2 to 4 s (Ekman, 2003b) and can be easily identified by humans. Over the past few decades, many researchers have made their efforts to help computer better understand facial expressions and the form of emotional communications among humans (Fasel and Juergen, 2003; Zhang and Tjondronegoro, 2011; Li X. et al., 2013; Li Y. et al., 2013). However, psychological studies (Porter and Ten Brinke, 2008; Ekman, 2009) indicate that the recognition of human emotion based on facial expressions may be misleading. In other words, someone may try to hide their emotion by exerting an opposite facial expression.

As a special facial expression, micro-expression is defined as a rapid facial movement that is not subject to people's consciousness and can reveal the genuine emotion (Ekman, 2003a). Micro-expression was first discovered by Haggard and Isaacs (1966), they found the Micro-expression is related to self-defense mechanism and can reveal depressed emotions. In 1969, Ekman and Friesen also observed a specific kind of micro-expression when they were analyzing a video from a depressive patient who attempted to tell a lie to cover his suicidal intent. In that video, the patient was optimistic by observing his facial expression, but when the video was played in a slower speed and inspected frame by frame, Ekman et al. saw an intense expression of extreme anguish just within two frames as the patient was answering a question from the doctor. The short expression lasted <1/12 s. From then on, understanding and recognizing micro-expression became a popular research topic (Russell et al., 2006; Endres and Laidlaw, 2009; Pfister et al., 2011).

For its authenticity and objectivity, micro-expression recognition possesses great value in diverse fields, such as, affect monitoring (Porter and Ten Brinke, 2008), criminal detection (Russell et al., 2006), and homeland security (Weinberger, 2010). However, due to its characteristics, micro-expression recognition is very challenging. Firstly, micro-expressions are fleeting and imperceptible, which typically last <1/2 s and can be easily neglected by human eyes (Yan et al., 2013a). Secondly, its intensity is very subtle and localized (Porter and Ten Brinke, 2008), i.e., micro-expression is a tiny movement confined to a small area of the face region. In 2009, Frank et al. found that only highly trained individuals are able to distinguish various micro-expressions, but the recognition accuracy is just 47%.

### Related research works

For the reason of the difficulty for human to notice or recognize micro-expressions, in recent years, automatic facial micro-expressions recognition has attracted increasing attentions in both the field of pattern recognition and computer vision (Polikovsky et al., 2009; Pfister et al., 2011). Polikovsky et al. (2009) presented a 3D-Gradient orientation histogram descriptor to represent the motion information in facial micro-expressions. Shreve et al. (2011) proposed a spatio-temporal strain method for automatic micro-expression spotting in long-term videos. Wu et al. (2011) designed an automatic micro-expression recognition system by using Gabor feature and GentleSVM classifier.

Thanks to Pfister et al. (2011), Li X. et al. (2013), Yan et al. (2013b, 2014), three spontaneous micro-expressions database (SMIC, CASMEI, and CASMEII) were built in well designed and strictly controlled laboratory environment and publicly introduced to the community. A brief summary of these three databases are given in Table 1. Based on the spontaneous database, many methods for micro-expression recognition have been proposed. Pfister et al. (2011) performed the first successful attempt in spontaneous facial micro-expression recognition. By Combining the Local Binary Pattern on Three Orthogonal Planes (LBP-TOP) descriptor and Random Forest (RF) classifier, the best accuracy of 78.9% on the SMIC database was obtained. Considering the redundant information in LBP-TOP features, Wang et al. (2014) proposed a LBP-Six Intersection Points (LBP-SIP) method and the experiment on CASMEII database shows that the LBP-SIP is more accurate and computational efficient than LBP-TOP. Huang et al. (2016) considered more information such as, sign, magnitude and orientation and proposed Spatiotemporal Completed Local Quantization Patterns (STCLQP) for facial micro-expression analysis. Compared with the LBP-TOP and LBP-SIP methods, STCLQP achieves a substantial improvement on recognition rate tested on the three public spontaneous micro-expression databases. Aside from concentrating on Spatiotemporal Local Texture Descriptors (SLTD) based methods, a more comprehensive research is done by Liu et al. (2015). In their work, a simple but efficient method called Main Directional Mean Optical-flow (MDMO) was employed, which utilized optical flow estimation technique to compute the subtle movement of facial regions of interest (ROIs) that were spotted based on the Facial Action Coding System (FACS). For 36 ROIs, the length of a MDMO feature vector is just 72. Besides, they also proposed an optical-flow-driven method to align all frames of a micro-expression video clip. To address the problem of constant head movements in typical micro-expression applications, Xu et al. (2017) presented Facial Dynamics Map (FDM) to characterize micro-expression. Based on Facial Landmark Location, “Coarse Alignment and Face Cropping” were conducted on the raw micro-expression clips, then a pixel-level alignment method was applied before FDM feature extraction. By classifying more categories and taking a different measuring method of recognition rate, the recognition accuracy on three databases (SMIC, CASMEI, and CASMEII) are 71.43, 42.02, and 41.96%, respectively.

**Table 1:** Three main spontaneous micro-expression database.

	**Index**			
	**Clips number**	**Camera speed**	**Frame size**	**AU coding/Labeling**
SMIC	164	100 fps	640 × 480	No/By Emotion
CASME I	195	60 fps	Part A: 1280 × 720	Yes/By Emotion
			Part B: 640 × 480	
CASME II	247	200 fps	640 × 480	Yes/By Emotion

The aforementioned works make solid contribution in automatic micro-expression recognition and inspire the community. However, there is still space to improve the methods. Firstly, the methods rely excessively on hand-crafted features and the process of feature selection depends heavily on the experience of researchers, which makes it difficult for psychologist lack of such experience to use the methods. Secondly, the recognition rate of the methods is not high enough for practical applications. Therefore, a more effective method that can generate high-level feature automatically for micro-expressions recognition is desired.

### Related research works

Convolutional Neural Networks (CNNs) (LeCun et al., 1998), as an effective deep learning model, has recently made unprecedented progress in many fields such as, computer vision (Szegedy et al., 2015), speech recognition (Abdel-Hamid et al., 2012), and natural language processing (Sutskever et al., 2014). Some popular CNN models like LeNet-5 (LeCun et al., 1998), AlexNet (Krizhevsky et al., 2012), GoogLeNet (Szegedy et al., 2015), and VGG-Net (Simonyan and Zisserman, 2014a) are well tested and widely used by many researchers. In spite of the difference in network structure, these popular deep networks are all shown their powerful ability for understanding the property of raw data. Except for 2D information processing, Karpathy et al. (2014) extended the connectivity of CNN to time domain and introduced a video descriptor to learn the spatio-temporal information. In the experiment on UCF101 Action Recognition dataset that contains 1 million YouTube videos belonging to 487 classes, the best recognition rate reached 63.9%.

In those successful works of CNN, large dataset is needed to train the network. However, the micro-expression database that we can use so far is much smaller than traditional database fed to CNN. A serious overfitting problem would occur if we directly apply CNN on the existing micro-expression database. In this paper, The proposed Dual Temporal Scale Convolutional Neural Network (DTSCNN) addressed the overfitting problem from three aspects: (i) the feature extraction was done on the micro-expression clips by using two shallow network separately; (ii) data augmentation and higher drop-out ratio were used in each network; (iii) CASMEI and CASMEII database were used together to train the network.

Meanwhile, the shallow network of DTSCNN has the risk of only learning low-level features. To ensure the proposed architecture can obtain high-level features, the data fed to the network was not raw data but the optical-flow, which is higher level feature than raw data and has been proved to be effective in micro-expression recognition (Liu et al., 2015).

The proposed DTSCNN is a two-stream convolutional network, each stream is a simplified network that uses 3D convolution kernel and pooling cell (Tran et al., 2015) to automatically represent the property of subtle facial movements. Because the frame rates of the video clips in CASMEI and CASMEII were 60 and 200 fps, respectively. One stream of the DTSCNN took 64 fps input (64 = 2^6^ adapts to CASMEI), and the other stream took 128 fps input (128 = 2^7^ adapts to CASMEII). Neither do we need the sophisticated frame alignment method nor the complicated feature design. The DTSCNN takes optical-flow sequences in different temporal scales as the input and outputs their higher level features. Experimental results on CASME I/II database demonstrate that our proposed method gave higher recognition rate than some state-of-the-art recognition methods, such as, STCLQP (Huang et al., 2016), MDMO (Liu et al., 2015), and FDM (Xu et al., 2017).

The following sections are organized as: section Convolutional Neural Networks gives a brief introduction of Deep learning (DL), and Convolutional Neural Network (CNN) principle; section Micro-Expression Recognition describes our proposed DTSCNN; section Experiments Results and Analysis presents and discusses the experiment design and results; section Conclusion gives the conclusion.

## Convolutional neural networks

In this section, we give a brief introduction of Deep learning (DL) and the Convolutional Neural Network (CNN) principle, which lays a foundation for proposing DTSCNN in section Micro-Expression Recognition.

### Deep learning

Deep learning is evolved from the research on neural networks. Typically, it is composed of multiple processing layers and has powerful abilities to learn representations of data using multiple levels of abstraction. Currently, many deep network structures have been put forward. Such as, Deep Belief Network (Hinton et al., 2006), Stacked Auto Encoders (Vincent et al., 2010), Convolutional Neural Network (LeCun et al., 1998), and Recurrent Neural Network (Mikolov et al., 2011). For the dramatically great success of CNN in visual object recognition and detection, in this paper, we mainly discuss the CNN for micro-expression recognition.

### Convolutional neural network (CNN)

**Figure 1:**
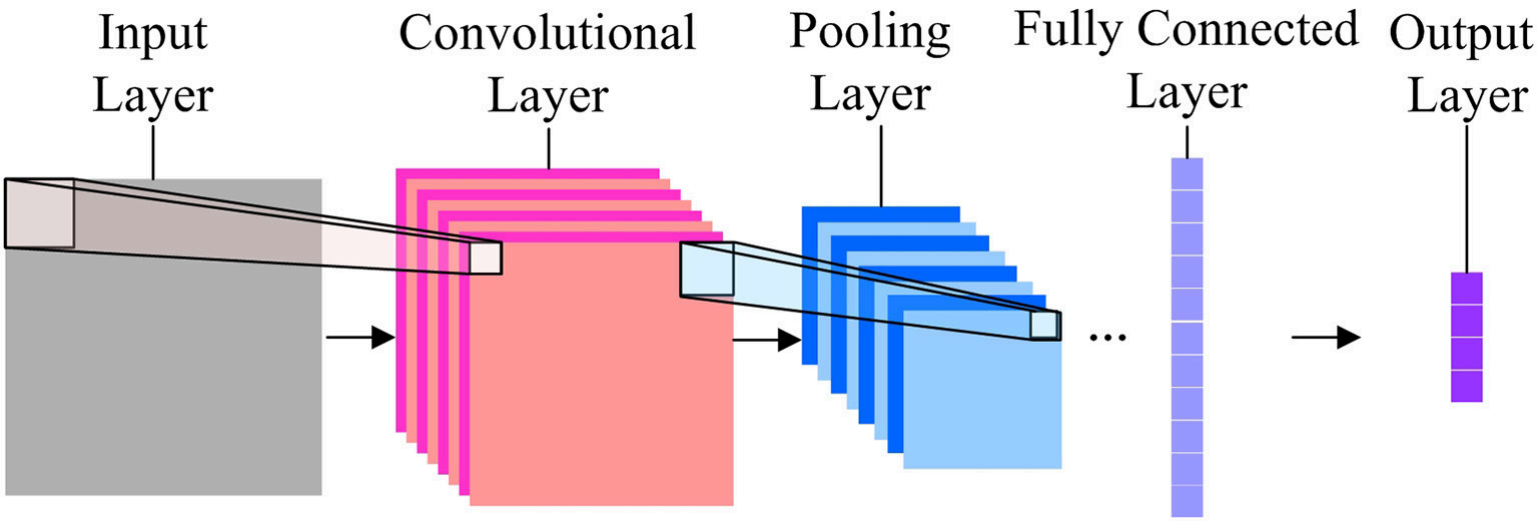
The General structure of a CNN.

CNN is a biologically-inspired model and firstly proposed by LeCun et al. (1998). Shown in Figure 1 is a general structure of a CNN.

In Figure 1, the input layer receives normalized images with identical size. A set of units in a small neighborhood (local receptive field) in the input layer will be processed by a convolution kernel to form the unit in a feature map of the subsequent convolutional layer. One pixel in the feature map can be calculated by using

(1) $$C_{k}=f(x*W+b)$$

where *C*_*k*_ is the value of the *k*-th pixel in the feature map, *x* is the pixel value vector of the units in the local receptive field corresponding to *C*_*k*_, *W*, and *b* are the coefficient vector and bias, respectively, determined by the feature map, while *f* is the activation function (sigmoid, tanh, ReLU, etc.). Since studies in Nair and Hinton (2010) have suggested that ReLU function is superior to sigmoid function, in our work, the ReLU function has been employed. For the input*t*, ReLU function can be expressed as

(2) f(t)=max(0,t)

Each feature map has only one convolutional kernel, i.e., for all *x* in the input plane, the *W* and *b* are the same. This design of CNN can largely save calculation time and make specific feature stand out in a feature map. There is normally more than one feature map in a convolutional layer, so that multiple features are included in the layer.

To make the feature invariant to the geometrical shift and distortion, the convolutional layer is followed by a pooling layer which can subsample the feature maps. For the *k*-th unit in a feature map in the pooling layer, its value can be calculated by using

(3) $$P_{k}=f(\beta *down(C)+\alpha )$$

where *P*_*k*_ is the value of the *k*-th unit in feature map in the pooling layer, *C* is the value vector in the feature map of the convolutional layer, β and α are the coefficient and bias, respectively, and *down*(•) is the subsampling function. Max pooling function is used for subsampling, in that case, *down* (*C*)can be written as

(4) $$down(C)=\max\;\left\{C_{s,l}|s\leq m,l\leq m,s,l\in z^{+}\right\}$$

where *C*_*s,l*_ is the pixel value of the unit *C* in the feature map, *m*is the subsampling size.

The first convolutional layer and pooling layer would acquire low-level information of the image, while the stack of them would enable high-level feature extraction.

The output layer is connected to its formal layer with Softmax Regression. For the output vector F from the upper layer, the probability of classifying into class c is:

(5) $$p\left(y^{\left(F\right)}=c|F;\theta \right)=\frac{e^{\theta_{c}^{T}F}}{\sum_{n=1}^{N}e^{\theta_{n}^{T}F}}\;1\leq c\leq N$$

where *y*^(*F*)^ is the group identity of input *F*, θ is weight vector between output layer and previous layer, *N* is the number of the groups. The loss function is defined as:

(6) $$J(\theta )=-\sum_{c=1}^{N}1\;\{y^{\left(F\right)}=c\}\;\log\frac{e^{\theta_{c}^{T}F}}{\sum_{n=1}^{N}e^{\theta_{n}^{T}F}}\;1\leq c\leq N$$

Where, {♦} is the eigenfunction, when {♦} is true, it will return 1. Practically, in CNN training, we would compute the sum of loss function from multiple inputs, and update the weight of network using stochastic gradient descent (Wilson and Martinez, 2003).

## Micro-expression recognition

### Pre-processing

At the stage of data pre-processing, two techniques are contained: face alignment and normalization. In face alignment, we take the method presented in Yan et al. (2014). In their method, 68 facial landmarks are detected in the first frame in each micro-expression video clips using Active Shape Model (ASM) (Cootes et al., 1995). Then the first frame of each sequence is normalized according to the alignment template, the subsequent frames in each clips are all aligned to the first frame by using Local Weighted Mean (LWM) transformation (Goshtasby, 1988). In normalization, we normalize the aligned micro-expression samples both in spatial and temporal domain. For spatial domain normalization, all images are cropped within face region to 96 × 112 pixels, which is in the average size of the original face region in the database. For temporal normalization, we employ the linear interpolation method to obtain a sufficient number of frames. The linear interpolation method is widely used and proved to be effective in frame normalization (Liu et al., 2015; Xu et al., 2017). As mentioned in the early section, the training set that we used contains two subsets, where video clips are normalized to 65 frames and 129 frames, respectively, to compensate for frame differences of those two databases.

### Optical flow estimation

Optic-flow technique can detect the motion information between adjacent frames. In analyzing visual motion information, optical flow is typically served as a high level feature in machine learning area. Recently, some large-scale video classification works with CNNS (Simonyan and Zisserman, 2014b; Tran et al., 2015) has also suggested that optical flow sequences are more efficient to use than the original image sequences.

In a video clip, suppose that *I*(*x, y, t*) is the value at point (*x, y, t*). After a lapse of δ*t* to the next frame, the pixel moved to (*x* + δ*x, y* + δ*y, t* + δ*t*) with its intensity *I* (*x* + δ*x, y* + δ*y, t* + δ*t*). Based on invariance of brightness during small period, we have

(7) I(x,y,t)=I(x+δx,y+δy,t+δt)

where δ*x* = *uδt*, δ*y* = *vδt*, with *u*(*x, y*) and *v*(*x, y*) to be the horizontal component and vertical component that need to be estimated in the optical flow field.

If we assume that the pixel value in an image is a continuous function of its position and time, according to the Taylor series expansion, the right part of the function (7) can be written as:

(8) $$\begin{array}{l} I\left(x+\delta x,y+\delta y,t+\delta t\right)=I\left(x,y,t\right)+\delta x\frac{\partial I}{\partial x}+\delta y\frac{\partial I}{\partial y}\\ \;+\;\delta t\frac{\partial I}{\partial t}+\varepsilon \end{array}$$

Where ε is the two order or above unbiased estimator of time δ*t*. When δ*t* tends to be infinitesimal, we can let both sides of formula (8) to be divided by time δ*t* and the Equation (7), then the optical flow equation is obtained as follows:

(9) $$\frac{\delta x}{\delta t}\frac{\partial I}{\partial x}+\frac{\delta y}{\delta t}\frac{\partial I}{\partial y}+\frac{\partial I}{\partial t}=0$$

that is,

(10) $$u\frac{\partial I}{\partial x}+v\frac{\partial I}{\partial y}+\frac{\partial I}{\partial t}=0$$

**Figure 2:**
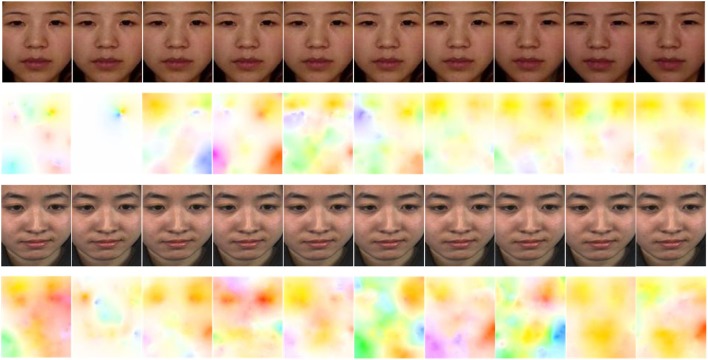
Two samples are pre-processed and estimated optical fields. The first row and third row micro-expression sequence are from CASMEI (subject 01, EP12_3, frames: 45–54) and CASMEII (subject 17, EP05_02, frames: 53–62) database, respectively. The former one expressed a surprise emotion and the latter expressed a positive emotion (happiness). Row 2 and 4 is the optical flow sequence that computed from their above line, respectively.

For video clips of micro-expression, computing the tiny movement of facial region accurately is crucial before recognition. In Liu's work (Liu, 2009), he made subtle movements in the video more obvious by computing its optical flow estimation, which is also suitable for us in order to recognize the micro-expression information. Further, the matrix of *u, v* fields can be transformed to image by using Munsell Color System (Gargi et al., 2000). Figure 2 shows two pre-processed samples and their optical field estimations from CASME I/II. To human eyes, it is hard to notice the facial change in those clips. However, in optical flow fields, we could demonstrate the subtle movement in different colors.

### DTSCNN

DTSCNN is a two streams network with 3D convolution and pooling units. Unlike typical convolution or pooling cell in convolutional neural network, the 3D convolution and pooling in DTSCNN have a kernel in size of k × k × l, where k is spatial size, l is temporal depth. The micro-expression clip that we refer to in DSTCNN has a size of d × w × h × c, where w, h, and c are width, height and number of channels of every single frame, respectively, and d is the number of frames.

In an input layer of a typical convolutional neural network, every single image is treated as an object to be identified. Nevertheless, in video classification, each video clip is used as a bag of words and fed into the network. In our work, we calculated the optical flow estimation in size of 64 × 96 × 112 × 3 and 128 × 96 × 112 × 3 for each micro-expression video clip in CASME I/II dataset.

**Figure 3:**
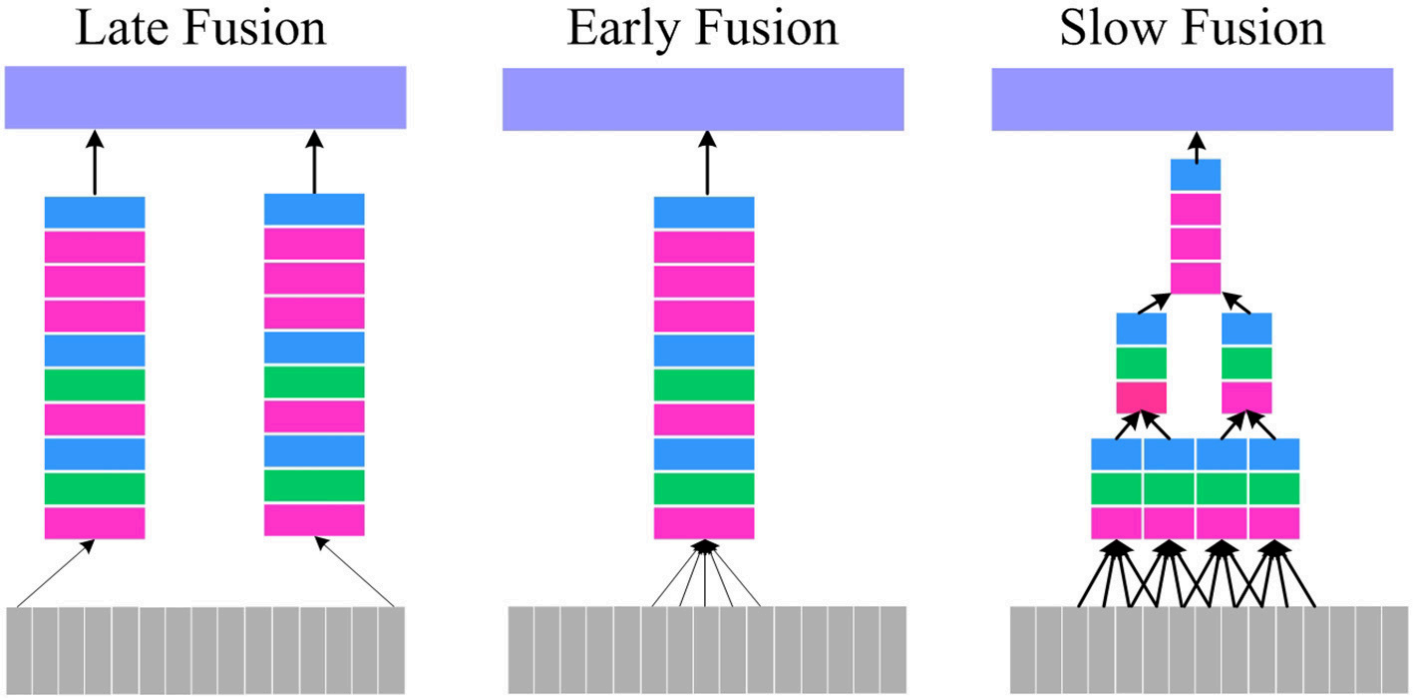
Fusion model. **Left**: Late Fusion. **Middle**: Early Fusion. **Right**: Slow Fusion. The red, green, blue, and purple box represent convolutional, pooling, normalization and Fully-Connected (FC) layers, respectively.

For continuous-time visual information processing, temporal information is as important as spatial information. However, how to probe the spatio-temporal information sufficiently and effectively is critical to video identification task. In Karpathy's work (Karpathy et al., 2014), three connectivity patterns of convolution neural network in video identification task were presented. Figure 3 shows these three kinds of fusion model.

In Figure 3, The Late Fusion model is similar to parallel convolutional neural network and each single-frame network shares parameters in a fixed frame distance. The Early Fusion model design is based on single-frame networks and only utilizes 3D convolution with a size of k × k × l in the first layer to extract the spatio-temporal information. The Slow Fusion model is a more comprehensive combination, which utilizes the 3D convolution and pooling technique throughout the network while learning more elaborate information from both spatial and temporal domains. Although this would progressively generate higher-level information, it is slow and memory-consuming.

**Figure 4:**
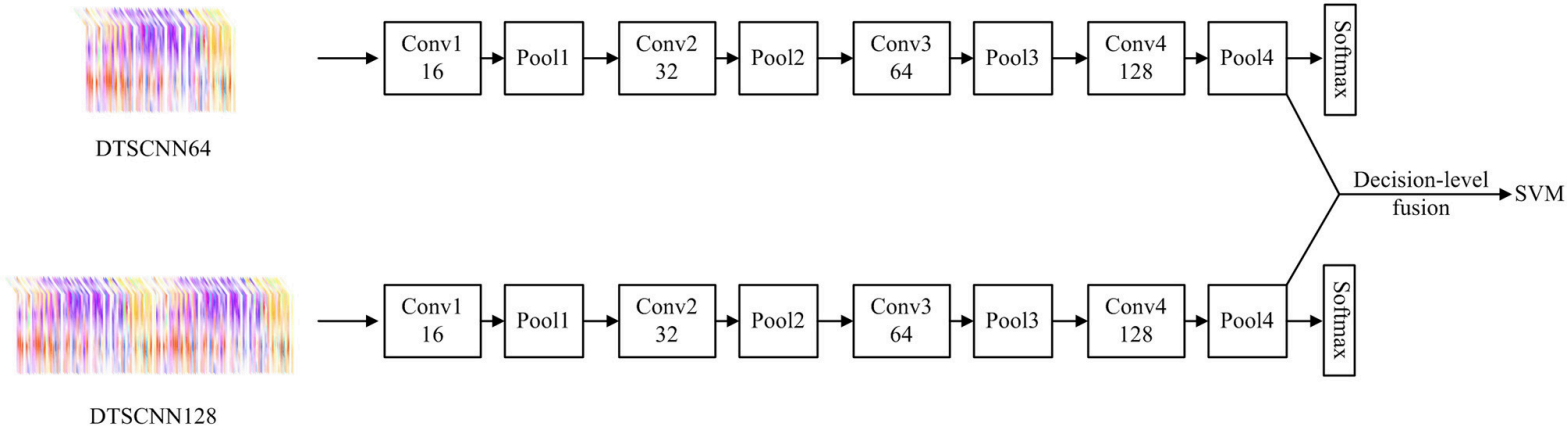
Dual Temporal Scale Convolutional Neural Network (DTSCNN).

For micro-expressions recognition, considering that the micro-expression is continuous and is not contained in a specific frame or few adjacent frames, the Late Fusion and Early Fusion may be inadequate. In addition, Karpathy's (Karpathy et al., 2014) and Du's (Tran et al., 2015) experiments show that Slow Fusion model can give better performance than Late and Early fusion model. Especially, Du et al proposed the C3D (Tran et al., 2015) and proved that a 3 × 3 × 3 convolution kernel used in every layer would give the best performance. Therefore, in our work, we combine the Slow Fusion model and C3D implement for micro-expression recognition. Specially, a DTSCNN is proposed and the architecture is shown in Figure 4.

In Figure 4, we can see that DTSCNN is a two-stream convolutional network consisting of DTSCNN64 and DTSCNN128. Each stream is compact with only 5 layers (4 convolutional layers and 1 fully-connected layer, the number of filters for the four convolution layers is 16, 32, 64, and 128, respectively. The detail of the kernel parameter setting of the network is given in Table 2. In the first convolution layer (3 × 3 × 8 conv or 3 × 3 × 16 conv), a big spatial and temporal stride is set to omit redundant information in that initial level and save memories. The setting of second and third layer (3 × 3 × 3 conv) follow Du's (Tran et al., 2015) conclusions. The reasons of the fourth layer utilizing 3 × 3 × 4 convolutional filter is that a 3 × 3 × 3 convolution filter may create more temporally indefinite factors when it operates previous layers that with 4-frames length. The last layer is an output layer since keeping an extra FC layer consumes time and memory. Using a two-stream architecture can not only overcome the frame rate difference between CASMEI and CASMEII but also the overfitting problem due to small data size. Also, taking the optical-flow data as input can help the simplified network to learn high-level feature. When learning is finished, a linear SVM classifier is used to take features from the final layer of each stream. The result of the SVM classifier is used for decision-level fusion to give the overall recognition rate.

**Table 2:** Parameters and size of the kernel in DTSCNN.

**Layer**	**DTSCNN64 (Kernel parameter settings)**	**DTSCNN128 (Kernel parameter settings)**
Input	–	–
Conv1	3 × 3 × 8, Sp:1,Ss:2,Tp:2,Ts:4	3 × 3 × 16, Sp:1,Ss:2,Tp:4,Ts:8
pool1	2 × 2 × 1, Ss:2,Ts:1	2 × 2 × 1,Ss:2,Ts:1
Conv2	3 × 3 × 3,Sp:1,Ss:1,Tp:1,Ts:1	3 × 3 × 3,Sp:1,Ss:1,Tp:1,Ts:1
pool2	2 × 2 × 2,Ss:2,Ts:2	2 × 2 × 2,Ss:2,Ts:2
Conv3	3 × 3 × 3,Sp:1,Ss:1,Tp:1,Ts:1	3 × 3 × 3,Sp:1,Ss:1,Tp:1,Ts:1
Pool3	2 × 2 × 2,Ss:2,Ts:2	2 × 2 × 2,Ss:2,Ts:2
Conv4	3 × 3 × 4,Sp:1,Ss:1,Tp:0,Ts:1	3 × 3 × 4,Sp:1,Ss:1,Tp:0,Ts:1
pool4	2 × 2 × 1,Ss:2,Ts:1	2 × 2 × 1,Ss:2,Ts:1
Classify	–	–

*Sp, Ss, Tp, and Ts denote spatial padding, spatial stride, temporal padding and temporal stride respectively*.

**Figure 5:**
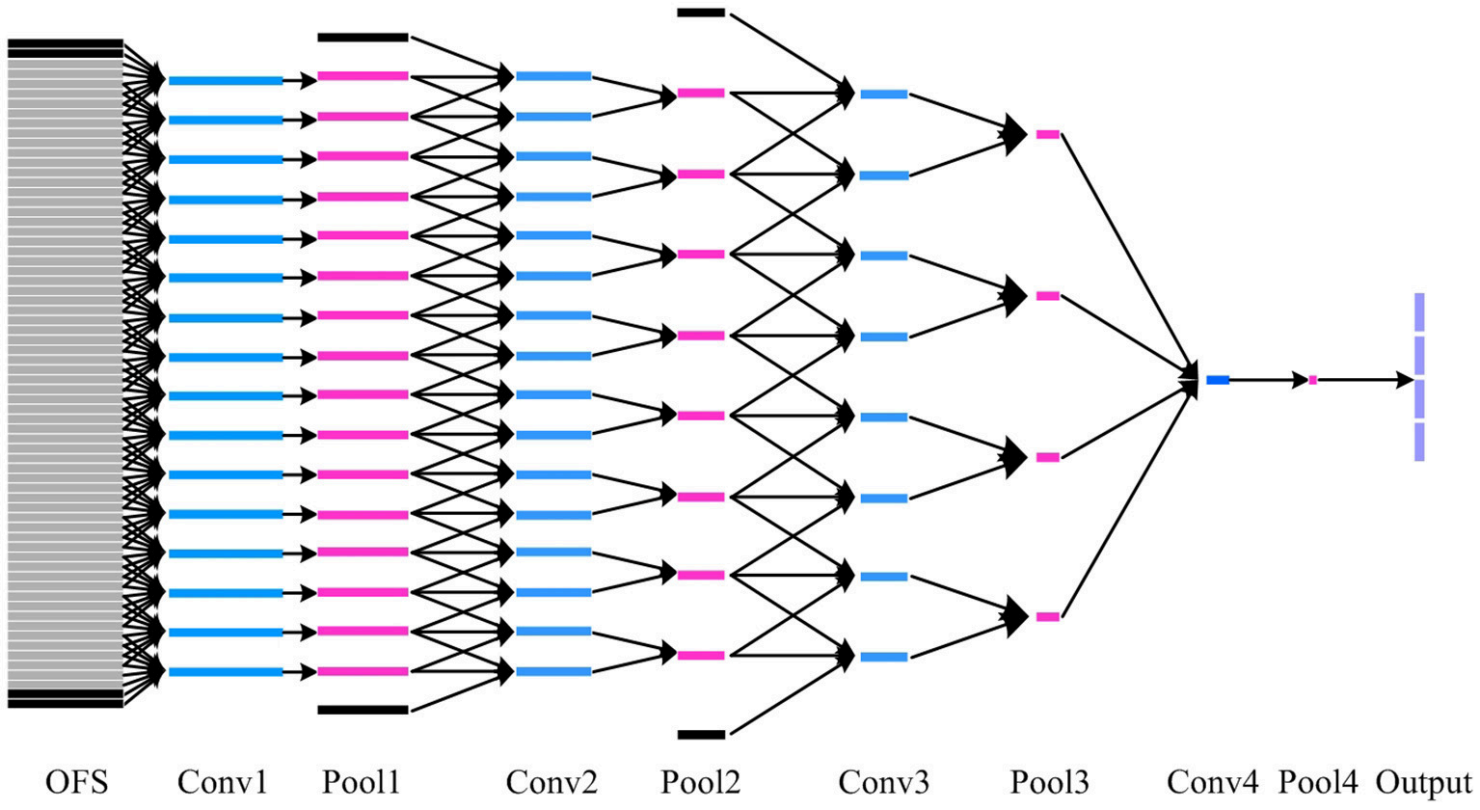
The details of how 3D convolutional kernel in DTSCNN64 process a video clip. The blue, pink and purple box represents convolutional, pooling, and classifying layers, respectively. OFS denote optical flow sequence. The black box indicates the padding image in temporal domain. Each gray box represents an optical flow image. Besides, all parallel blue boxes in the same layer share parameters.

The DTSCNN64 and DTSCNN128 are designed to take micro-expression video clips in size of 64 × 96 × 112 × 3 and 128 × 96 × 112 × 3, respectively. The DSTCNN64 is used to adapt to the frame rate of 60fps of CASMEI, and the DSTCNN128 to CASMEII. This design is important in real application. Because there is no agreed standard frame rate so far for recoding the micro-expressions, i.e., the micro-expression video could be recorded in various frame rate. The design of different streams of the network can adapt to different frame rates, which may make the whole network robust to the frame rate of the input data. The prediction falls into four different classes. Figure 5 shows the detail of DTSCNN64.

## Experiments results and analysis

### Database and experiment setting

In CASMEI database (Yan et al., 2013b), there are 189 spontaneous micro-expression video clips collected from 19 subjects. Each clip was filmed by a 60-fps camera with a size of 640 × 480 pixels. The data can be classified into 8 classes. Compared with CASMEI, CASMEII (Yan et al., 2014) is more like an updated version. Namely, it contains 255 spontaneous micro-expression video clips from 26 subjects and includes emotion belonging to seven classes. Especially, it was recorded by camera with a speed of 200 fps and the face region occupies a larger proportion in the image. In our experiment, we selected data from CASMEI and CASMEII to form the experiment dataset CASME I/II. Following the recommended strategy (Yan et al., 2013b, 2014), we categorized CASME I/II into four classes: Negative, Others, Positive and Surprise. The specific emotion that each class contains and the number of clips in CASME I/II are shown in Table 3.

**Table 3:** Specific emotions in each category and clip numbers in experiment database.

**CASMEI/II**	**CASMEI**	**CASMEII**
Negative (124)	Disgust (44), sadness (6), fear (2)	Disgust (63), sadness (7), fear (2)
Others (234)	Tense (69), repression (38), contempt (9)	Repression (27), Others (99)
Positive (41)	Happiness (9)	Happiness (32)
Surprise (45)	Surprise (20)	Surprise (25)

Currently, many methods have been tried on the spontaneous micro-expressions database. In this paper, we compare DTSCNN with three state-of-the-art methods, i.e., STCLQP (Huang et al., 2016), MDMO (Liu et al., 2015), and FDM (Xu et al., 2017). The 3-fold cross-validation was used for all the methods evaluated on CASME I/II dataset.

However, from Table 3 we can see that the size of training data in each fold of cross-validation is relatively small for DTSCNN. Another problem that may affect the classification task is imbalanced classification data (He and Garcia, 2009). In cross-validation, there exists some imbalance in our training set. To address the issue, Liu et al. (2015) applied polynomial SVM to evaluate the accuracy of the testing phase, Huang et al. (2016) and Xu et al. (2017) used F_1_ score as an important index to measure the identification performance.

**Figure 6:**
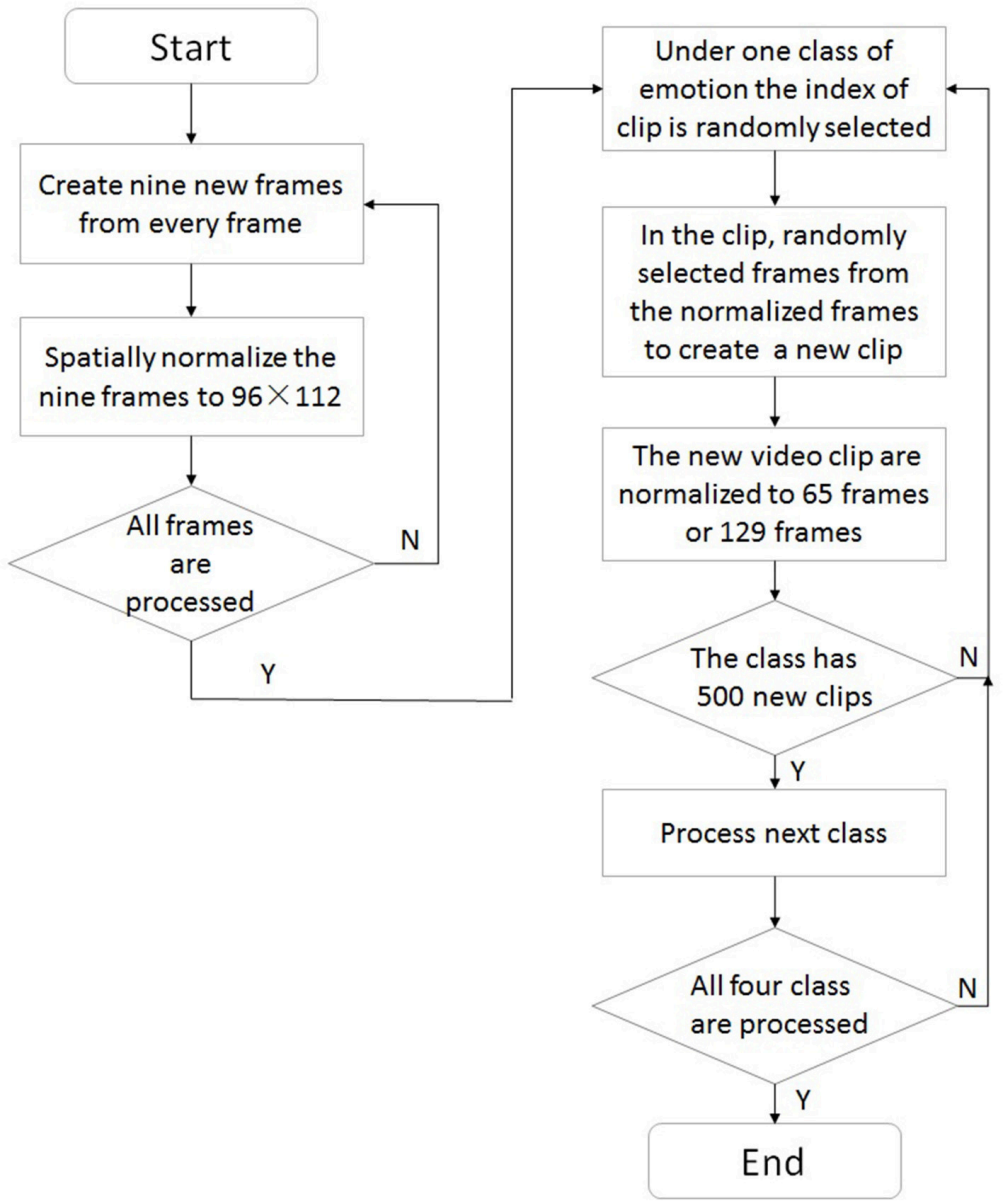
Flow chart of the sampling method.

In our work, we utilized a sampling method as a data augmentation strategy to solve both imbalanced learning and small-sample problems in each fold of the cross-validation. The sampling method is illustrated by using a flow chart in Figure 6 and conducted as following steps.

1. A slice of images with 2 pixels in width or height is cut from every frame in the CASMEI/II. By cutting at different places, i.e., up, down, left, right, center and upper left, upper right, lower left and lower right part of the frame, nine new frames can be created.
2. The created nine frames are spatially normalized to 96 × 112 pixels.
3. Repeat step 1 and 2 till all frames in the CASMEI/II are processed.
4. For one class of emotion, the index of video clip is randomly selected, suppose the j-th video clip is selected.
5. In the j-th video clip, replace every original frame with the frame randomly selected from its corresponding nine spatially normalized frames to create a new video clip.
6. The created video clip are normalized to 65 frames if it is in CASMEI or 129 frames if it is in CASMEII by using linear interpolation method.
7. Repeat step 4 to 6 until 500 video clips are created in this class of emotion.
8. Repeat step 4–7 until every class of four classes has 500 video clips.

Finally, for each training set, we have 20,000 clips in total (4 × 500), the number of video clips in each test set remained unchanged.

### Experimental results on CASME I/II database

Table 4 shows the micro-expression recognition accuracy of DTSCNN compared with the three methods. The experimental details of DTSCNN, STCLQP, MDMO, and FDM are as follows.

**Table 4:** The micro-expression recognition results (%) on CASMEI/II dataset with different methods, the fusion in bracket denotes the result is computed after done the decision-level fusion using Equation 10.

**Methods**	**Fold1**	**Fold2**	**Fold3**	**Average**
DTSCNN64 TIM64	65.45	65.45	65.45	65.45
DTSCNN128 TIM128	65.45	66.36	65.45	65.75
DTSCNN (fusion)	67.27	67.27	65.45	**66.67**
STCLQP TIM64	56.36	55.45	52.73	54.85
STCLQP TIM128	57.27	53.64	53.64	54.85
STCLQP (fusion)	58.18	56.36	54.55	**56.36**
MDMO TIM64	54.54	52.73	52.73	53.33
MDMO TIM128	54.54	54.54	53.63	54.24
MDMO (fusion)	57.27	55.45	53.63	**55.45**
FDM TIM64	53.64	53.64	54.55	53.94
FDM TIM128	53.64	53.64	55.45	54.24
FDM (fusion)	57.27	57.27	56.36	**56.97**

#### DTSCNN

The detail of the parameter setting of DTSCNN is given in Table 2. In the training phase, each stream employed batch gradient descent with a momentum of 0.9 (Wilson and Martinez, 2003), the dropout ratio of the FC layer was set to 0.5 and the minimum batch size was set to 10. The initial learning rate was set to 0.0001 and would get divided by 10 after every 10 epochs. Each stream of DTSCNN was trained separately, and the output feature of the Pool4 layer from each stream was fused for classification using linear SVM which would take decision-level fusion method to obtain final classification results. For each stream, if a sample x is classified into class C1 and class C2 with possibility of P1 and P2, respectively, then a decision-level fusion result C would be computed as follows

(11) $$C=\{\begin{array}{c} C1\;if.P_{1}\geq P_{2}\\ C2\;if.P_{1}<P_{2} \end{array}$$

#### STCLQP

Firstly, each video clip in CASME I/II database was given the same treatment as mentioned in section Pre-processing. Then, STCLQP feature extraction method that presented in Huang et al. (2016) was applied to each of the preprocessed sample. Finally, we used a SVM classifier with polynomial kernel (Schölkopf and Smola, 2002) to perform the classification. The parameters of SVM were refined using grid searching (Hsu et al., 2003).

#### MDMO

Prior to analyze the MDMO feature extraction methods, some processing steps which slightly different with the original paper (Liu et al., 2015) must be clarified. In particular, each raw video clip in CASMEI and CASME II database was given the same normalization treatment as mentioned in section Pre-processing. The following 36-ROIs detection of the first frame in each video clip was completed by using ASM model (Cootes et al., 1995) and FACS (Ekman and Friesen, 1977). Then, the optical flow sequence for each normalized samples were computed in the way mentioned in section Optical Flow Estimation. Finally, for each video clip, we obtained two optical-flow sequences in size of 64 × 480 × 640 × 3 and 128 × 480 × 640 × 3. In feature extraction and classification stage, the MDMO method was applied to each optical flow sequence to extract features from 36 ROIs, while a SVM with Gaussian kernel (Schölkopf and Smola, 2002) served as the classifier to evaluate the feature extraction performance.

#### FDM

For each video clip in CASME I/II, firstly, we took the pretreatment method mentioned in section Pre-processing. Then, the optical flow computation and FDM feature extraction step (Xu et al., 2017) were conducted. Finally, we used a Linear SVM classifier to evaluate the accuracy of the feature.

As shown in Table 4, an average accuracy of 66.67% is achieved by DTSCNN, which is higher than every single stream network (DTSCNN64: 65.47%, DTSCNN128: 65.75%), and outperforms all the traditional feature extraction based method (STCLQP: 56.36%, MDMO: 52.12%, FDM: 56.97%). Particularly, the recognition accuracy of DTSCNN is almost 10 percent higher than STCLQP, MDMO, and FDM.

**Figure 7:**
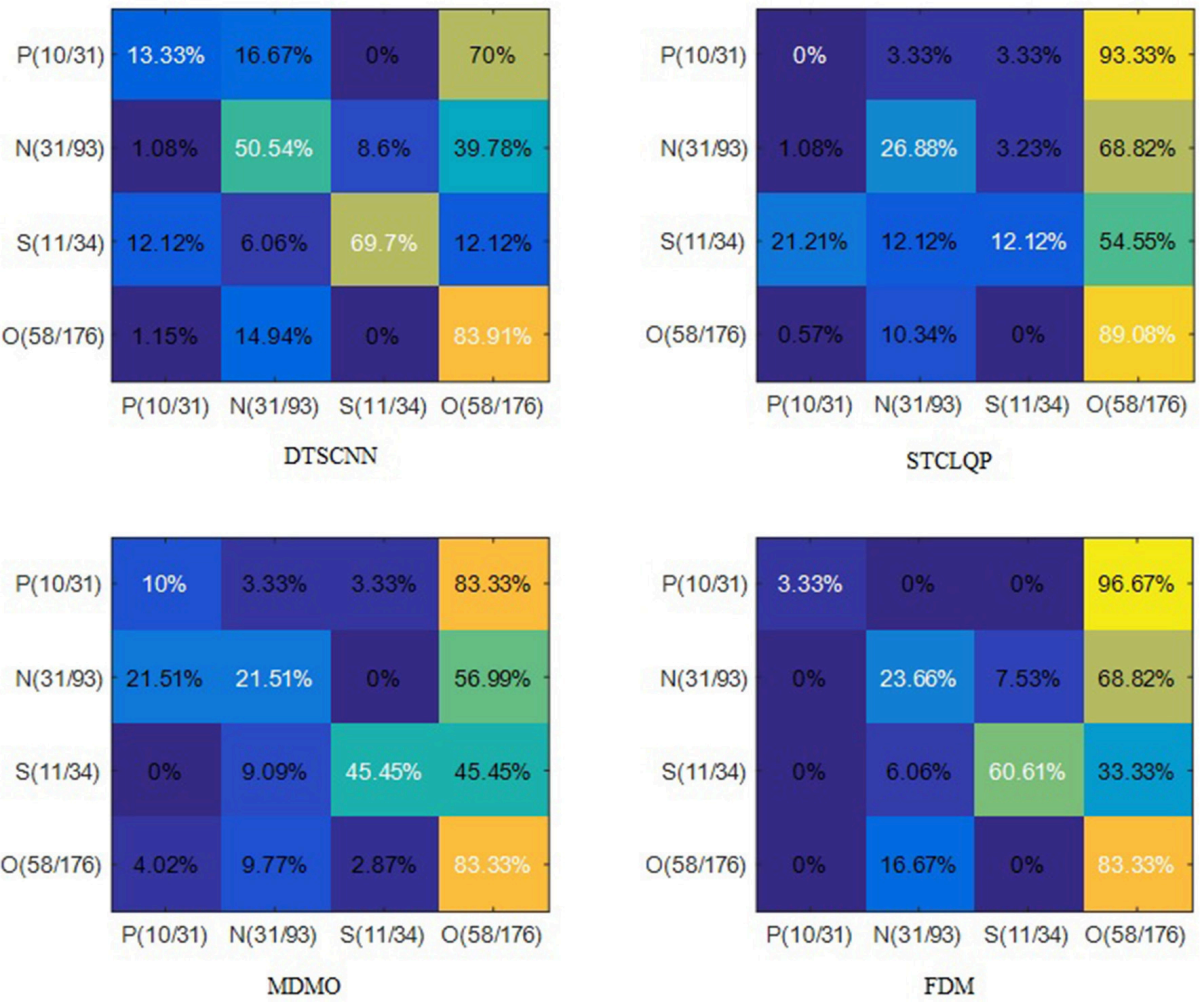
Confusion matrices on CASMEI/II dataset. The N, O, P, and S denote the classes of Negative, Others, Positive, and Surprise, respectively. The number in packets indicates the number of samples in testing set vs. the training set.

Figure 7 shows the average confusion matrices of the four methods. Apparently, the prediction of traditional methods would prefer the class with larger number of samples. For example, all three methods predict “Negative” as “Others” with chance of more than 55%, because “Other” class has larger training set. However, DTSCNN is robust to this imbalance-data effect, it can still predict “Negative” as “Negative” with chance of 50.54%. The good performance of the DTSCNN may be due to the sampling method employed by DTSCNN to address the problem of imbalanced data.

Among traditional methods, FDM is more robust to imbalanced-data effect. In predicting “Surprise,” only FDM can predict it with higher chance (60.61%), STCLQP and MDMO predict it as “Others” with chance of 54.55 and 45.45%, respectively.

DTSCNN has almost the highest rate of correct prediction according the confusion matrices (except in predicting “Others”). Especially in recognizing “Negative,” the DTSCNN has a correct prediction rate of 50.54%, which is more than 20% higher than those of STCLQP, MDMO, and FDM (26.88, 21.51, and 23.66%, respectively).

The main reason for the low recognition rate of “Positive” for all methods is due to very limited training samples (only 31). Nevertheless, the proposed DTSCNN method still archives the highest recognition accuracy rate of 13.33%.

To sum up DTSCNN can not only effectively learn features from imbalanced data, but also interpret the subtle movement in facial micro-expression clips internally and give an outstanding performance for quandary classification with negative, others, positive, and surprise.

## Conclusion

In this paper, we proposed a DTSCNN architecture to recognize spontaneous micro-expression. The DTSCNN is a simplified design and end-to-end trainable two-stream network. Specifically, each convolution and pooling cell is a 3D structure that employs the Slow Fusion model mechanism to process micro-expression sequence internally, while the two-stream architecture is designed to take sequences normalized to 64 frames and 128 frames separately so that more discriminative features can be learned from data in different temporal length.

In pretreatment, unlike traditional methods that take complicated processing to obtain better recognition performance, we took much simpler method. The first step was to align clips to their first frame. The second was to calculate the optical flow estimation from the aligned and normalized samples.

In the experiment, we tested the DTSCNN on CASME I/II dataset. Unlike the traditional hand-crafted feature based method, which is labor-expensing and time-consuming, the DTSCNN can automatically learn features from simply pre-processed samples and complete the classification for recognition. Experimental results demonstrated that the proposed method can achieve highest recognition rate among STCLQP, MDMO, and FDM. This also suggests that our proposed DTSCNN could be a promising method for micro-expression applications.

## Author contributions

MP and CW performed the data analysis, TC conceived the research, all authors wrote and read the article.

### Conflict of interest statement

The authors declare that the research was conducted in the absence of any commercial or financial relationships that could be construed as a potential conflict of interest.
